# A Numerical Thermo-Chemo-Flow Analysis of Thermoset Resin Impregnation in LCM Processes

**DOI:** 10.3390/polym15061572

**Published:** 2023-03-22

**Authors:** Hatim Alotaibi, Chamil Abeykoon, Constantinos Soutis, Masoud Jabbari

**Affiliations:** 1 Department of Mechanical, Aerospace and Civil Engineering, The University of Manchester, Manchester M13 9PL, UK; 2 Northwest Composites Centre, Department of Materials, The University of Manchester, Manchester M13 9PL, UK; 3 Aerospace Research Institute, The University of Manchester, Manchester M13 9PL, UK; 4 School of Mechanical Engineering, University of Leeds, Leeds LS2 9JT, UK

**Keywords:** liquid composite moulding, CFD, filling stage, curing stage, cure kinetics, rheology

## Abstract

This paper presents a numerical framework for modelling and simulating convection–diffusion–reaction flows in liquid composite moulding (LCM). The model is developed in ANSYS Fluent with customised user-defined-functions (UDFs), user-defined-scalar (UDS), and user-defined memory (UDM) codes to incorporate the cure kinetics and rheological characteristics of thermoset resin impregnation. The simulations were performed adopting volume-of-fluid (VOF)—a multiphase flow solution—based on finite volume method (FVM). The developed numerical approach solves Darcy’s law, heat transfer, and chemical reactions in LCM process simultaneously. Thereby, the solution scheme shows its ability to provide information on flow-front, viscosity development, degree of cure, and rate of reaction at once unlike existing literature that commonly focuses on impregnation stage and cure stage in isolation. Furthermore, it allows online monitoring, controlled boundary conditions, and injection techniques (for design of manufacturing) during the mould filling and curing stages. To examine the validity of the model, a comparative analysis was carried out for a simple geometry, in that the numerical results indicate good agreement—3.4% difference in the degree of cure compared with previous research findings.

## 1. Introduction

### 1.1. Background

**Figure 1:**
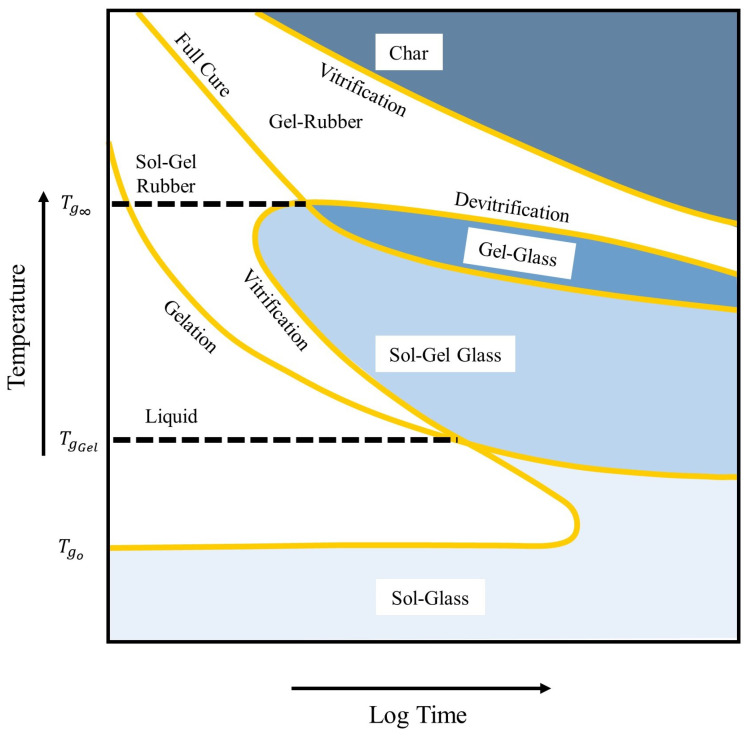
A schematic diagram for time–temperature–transformation (TTT) cure of resin systems [8,9].

Characterisation and modelling of thermoset resin impregnation (into fibrous preforms) in liquid composite moulding (LCM), e.g., resin transfer moulding (RTM), is of extraordinary importance in optimising such light-weight, high-specific stiffness and strength, and the durability of the produced composite structures. Flow mechanics and heat transfer that take place in LCM manufacturing processes are sensitive, complicated, and difficult to visualise by a fundamental method (trail-and-error experiments), which confines the knowledge and understanding of critical flow parameters such as the degree of conversion (cure), rate of reaction, development of viscosity, permeability, and capillary—resin–fibre wettability—effects. The optimisation of the aforementioned are even more important in the event of high-pressure injections or thick composite structures due to the high potential of voids or dry spot formation, as well as the non-uniformity of the cure degree during the manufacturing process. Furthermore, the mould design variables such as the locations and sizes of gates and vents and cavity geometry shapes play a significant role in the impregnation stage, and hence affecting the quality and the productivity of the final product [1,2]. It is therefore vital to develop a rigorous numerical model to predict cure kinetics and the chemo-rheology of such a complex thermosetting resin system during the infiltration of fabrics (fill) and cure stages. A numerical simulation of resin transfer (mould-filling) solves a moving boundary problem by engaging multiphysical paradigms wherein resin-flow, heat-transfer, and chemical (polymerisation) reaction occur concurrently. Lagrangian (or Arbitrary Lagrangian–Eulerian (ALE)) moving mesh (grid cells) and Eulerian fixed mesh are common methods to track and model free-surface flows using a numerical technique (e.g., volume of fluid coupled with finite volume method (FVM/VOF)) that converts a scalar transport equation into an algebraic equation for a feasible solution [3,4]. The Eulerian approach has received considerable attention in the literature for its simplicity and wide applicability, as it effectively describes flow motion by a volume fraction of the fluid within a fixed computational domain as well as avoids mesh regeneration—a large calculation time, while the Lagrangian or ALE involves moving and deforming domains, and hence the regeneration of the mesh [3,4]. Twinning energy (thermal) and species (chemical) equations with flow equations is imperative to model non-isothermal and reactive mould-filling process for advanced transport phenomena. The polymerisation (cure reaction) of the liquid resin occurs with cross-linking or chemical reactions to form a polymer matrix (i.e., three-dimensional (3D) networks) [5,6,7]. This will transform the liquid resin into a gelation stage and then to a solid state (glassy material), in which the shape cannot be remodified. Figure 1 explains the different stages of cure and gelation and their effects for thermosetting systems; this is illustrated by the so-called time–temperature–transformation (TTT) diagram [8,9]. During polymerisations, gelation and vitrification points are amongst the crucial transformations that could affect the quality (e.g., voids, shrinkage, thermal residual stresses, etc.) of the final product if not analysed or predicted by the traditional experimental tests or an advanced numerical software. The gelation point would highlight the required maximum pressure to be applied, while the vitrification point provides information about the ultimate reaction that could be reached (glass transition temperature equals reaction temperature) and the initiation of the glass transition process—for the formation of a rigid glass.

The thermosetting resin can undergo curing during the mould filling phase due to external effects such as the excessively high set mould temperatures, inappropriate mould designs, or low permeable zones. It is significant to have a processing window for the filling and curing stages that include the maximum cycle time, lower and maximum temperature, lower and maximum flow rate or pressure injections, and resin gelation and degradation. These processing parameters can be analysed by characterising the chemo-rheological behaviour of a thermosetting resin by means of practical methods [10,11,12,13,14,15], theoretical models [10,11,16,17,18,19], or numerical tools [20,21,22,23,24,25,26]. One of the prominent methods used to study the curing kinetics and the cross-linking reactions is the well-known differential scanning calorimetry (DSC) device, where an isothermal or non-isothermal analysis can be employed. This could provide exothermic and endothermic transformation data based on the enthalpy changes of a material system. It is worth mentioning that dielectric [27,28], ultrasonic [29,30], and fibre-optic [31] sensing methods have been effective applications for monitoring a cure in composites manufacturing. As a case in point, the dielectric system forms an electric field by the embedded copper electrodes to migrate ions, and such a mobility/conductivity will be affected by the viscosity development (cure) of the liquid resin [27,28]. Therefore, a change in ion conductivity can be used in the online monitoring of the curing in liquid moulding of composites. In this section, a theoretical background on cure kinetics and rheological behaviour is presented.

The curing characterisation of a thermosetting resin is typically required prior to the mould filling process of any kind of LCM processes (e.g., RTM, vacuum-assisted RTM (VARTM), etc.). The polymerisation reactions (of liquid resin samples “neat resin”) can be obtained by employing a number of experimental testing techniques including DSC and the Fourier transform infrared spectroscopy (FTIR). Dynamic (non-isothermal) experiments provide information on kinetic parameters together with the glass transition and crystallisation temperatures. These sets of information can also be provided by a series of isothermal experiments, in which a polymer sample is set to a predefined temperature. In general, the degree of cure or the extent of cure (α) is defined by the ratio of reaction heat $(\Delta H_{t}$) at a curing time (t) and the total (ultimate) heat of reaction $(\Delta H_{tot}$) as shown in Equations (1) and (2). This is obtainable during the exothermic process of a thermosetting resin sample in DSC or other differential thermal analysis (DTA) methods.

(1) $$\alpha =\frac{\Delta H_{t}}{\Delta H_{tot}}=0$$

(2) $$\alpha =\frac{1}{\Delta H_{tot}}\int_{0}^{t}\left(\frac{dH_{t}}{dt}\right)dt$$

(3) $$\frac{d\alpha }{dt}=\frac{1}{\Delta H_{tot}}\left(\frac{dH_{t}}{dt}\right)$$

The rate of reaction $(\frac{d\alpha }{dt}$) can then be given by rearranging/rewriting Equations (1) and (2) to be proportional to the rate of heat generation as given in Equation (3). This formulation form could provide information on the reaction or polymerisation rate of a polymeric material and can also be used to assess the process cycle time (long/short). In recent decades, researchers have developed empirical models to calculate the degree of cure and the rate of reaction for various thermosetting resins such as epoxy [11,12,13,17], and polyesters [16,32,33,34]. These models can be useful for numerical modelling tools to quantify viscosity variations as a function of cure and the applied temperature for a mould filling process of fibre-reinforced composites. Cure kinetics models are typically classified into two categories: (i) mechanistic and (ii) phenomenological. The mechanistic model was first introduced by Stevenson [35] based on a free radical polymerisation approach and this includes a number of stages as initiation, inhibition, propagation, and termination of a polymer material. However, such complexity motivated researchers [17,18,32,33,36] to simplify this approach by neglecting termination reactions and disconnect other polymerisation reactions. On the other hand, for more simplicity and maintaining the level of accuracy, the phenomenological models ($n^{th}$ and autocatalytic) proved their applicability in a wide range of thermosetting resins. This type ignores the details of a polymer cure reactions, so it assumes one equation to represent/describe the entire curing reaction. The phenomenological models for cure kinetics involve rate constants $\left(k_{1}\right)$ and $\left(k_{2}\right)$ that follow an Arrhenius behaviour. Therefore, the kinetic data (e.g., heat flow vs. time) must be determined via thermal analysis experiments. This would allow obtaining the rate of reaction and degree of cure data for different isothermal temperatures. With the nonlinear regression method, the kinetic rate constants together with the reaction orders (m) and (n) can be determined. Since this semi-empirical model obeys the Arrhenius law, a linear fitting plot, in other words, the linear relationship between the rate constants values with their isothermal temperatures give useful details of a liquid resin, that is, the activation energy $\left(E_{a}\right)$. While the $n^{th}$ order kinetics show a simple empirical solution with only one kinetic rate constant, it is still not applicable for thermosetting systems that normally achieve the maximum reaction rate at an intermediate extent of cure [37,38]. Thus, the autocatalytic kinetics model proposed by Kamal and Sourour [10,39] can sufficiently quantify curing processes for such resin systems, and hence it is also adopted in this work. Kamal and Sourour [10,17,39] used DSC to study the curing parameters for a variety of thermosetting systems (e.g., unsaturated polyester, and epoxy resins). They contributed with the well-known Kamal model (presented in Equation (4)) to calculate the isothermal rate of reaction, wherein the model agreed well with the populated experimental data

(4) $$\frac{d\alpha }{dt}=\left(k_{1}+k_{2}\alpha^{m}\right){\left(1-\alpha \right)}^{n}$$

(5) $$k_{1}=A_{1}\exp\left(\frac{-E_{a_{1}}}{RT}\right)$$

(6) $$k_{2}=A_{2}\exp\left(\frac{-E_{a_{2}}}{RT}\right)$$

where $A_{1}$ and $A_{2}$ are the pre-exponential constants, $\left(E_{a_{i}}\right)$ is the reaction activation energy, *R* is the universal gas constant, and *T* is the temperature. Under certain circumstances, a resin flow during the impregnation process experiences an increase in viscosity causing an earlier gelation. The mechanisms of gelation stem from different factors that include time, temperature, and chemical formulation [37,38]. Thus, resin systems with low viscosity, which is usually the case for LCM processes, undergo fast reaction (e.g., fast curing resins), leading to a nonlinear increase or evolution in the resin viscosity. The prediction of such a phenomenon can be achieved using rheological or the so-called chemo-rheological models [37,38]. This is required in order to avoid challenges relevant to the resin impregnation of fibre preforms. In some of the flow simulation tools (e.g., ANSYS Fluent, and OpenFoem), the semi-empirical models can be employed in an algorithmic code format to optimise or control viscosity variations during the fill and cure process cycle. Here, the commonly used model developed by Castro and Macosko [19] is applied in the present study. The Castro–Macosko model calculates the viscosity as a function of the degree of cure and temperature as given below in Equation (7).

(7) $$\mu \left(\alpha ,T\right)=\mu_{0}\exp\left(\frac{E_{\mu }}{RT}\right){\left(\frac{\alpha_{gel}}{\alpha_{gel}-\alpha }\right)}^{a+b\alpha }$$

where μ is the dynamics viscosity, $\mu_{0}$ is a pre-exponential factor, *a* and *b* are exponents, $E_{\mu }$ is the viscosity activation energy, and $\alpha_{gel}$ is the degree of cure at the gel point.

### 1.2. A State-of-the-Art Review

In an LCM process, such as RTM, the process cycle includes subsequent stages, i.e., filling/injection and curing. To optimise such a complex flow problem, the continuity and momentum, energy, and species equations should be solved simultaneously. Resin flow would be subject to effects by the degree of cure and temperature within the filling process, therefore, cure kinetics and chemo-rheological models need to be incorporated to capture flow behaviour trends, particularly viscosity variations, degree of cure, and rate of cure. When considering these aspects, two- or three-dimensional mould filling problems can be simulated more realistically. The numerical modelling of LCM processes has been an area of interest over the last few decades to meet industrial demands and to quantify relevant issues by applying innovative prediction methods via numerical simulations. Lee et al. [20] performed mould filling and curing simulation to characterise a thermoset type resin using finite element/control volume (FE/CV) method. They were able to capture the degree of conversion and temperature variation for a two-dimensional flow problem, which matched well with results from the experiments. Although temperature and curing histories were quantified by the simulation model [20], it was not applicable to 3D non-isothermal applications. Similarly, Bruschke and Advani [21] used FE/CV—implemented in liquid injection moulding simulation (LIMS) [40]—to solve free surface flows (flow-front) in porous media during the mould-filling stage. However, the authors [21] treated temperature and degree of cure (energy and species equations) with finite difference/control volume (FD/CV) approach—adopting Crank–Nicolson discretisation—in an attempt to provide a two-way coupling between the momentum and energy-species for addressing conduction (through-thickness) and convection (in-plane) in a three-dimensional form. Cheung et al. [22] implemented a coupled thermo-kinetic model using three-dimensional Galerkin finite element method. They numerically obtained the resin temperature and degree of conversion gradients during the cure process, in which a validation with an experimental date shows 3.89–5.7% error. It was claimed that large mesh problems could be quantified by the developed approach (element-free and iterative technique), however, the thermo-chemo kinetics formulation was limited to the curing phase. A study by Abbassi et al. [41] performed an implicit-FDM (finite difference method) numerical modelling of a thin rectangular mould for a RTM process. A heated porous fabric preform was proposed to analyse temperature changes and the conversion degree of the resin liquid impregnation throughout filling and curing phases. They highlighted the significance of the mechanical-heat dispersion term that should be considered to obtain the accurate temperature distribution profiles of resin impregnation/infiltration of a porous medium. The numerical solution [41] was merely dedicated to a decoupling iterative strategy—the separate calculations of flow fields (e.g., pressure, velocity, temperature, conversion degree). Leistner et al. [42] coupled the implicit Runge–Kutta with multilevel-Newton (DIRK/MLNA) methods based on a finite element approach to model the curing process of an epoxy resin sample placed in an aluminium container, in that, conservation equations for continuity and momentum was excluded (no flow motion). This was to quantify high-order time integrations (time-adaptive) and heat capacity and conductivity variables to numerically study the curing and glass transition temperature of the resin. It was, however, stressed that the evolution of temperatures was overestimated due to the inclusion of convection and radiation boundary conditions (b.c.) at the top surface of the liquid sample. An FEM-based PAM-RTM was applied by Poodts et al. [23] to simulate a resin flow within a fibrous porous medium for both filling and curing stages. The cure analysis was conducted assuming a fully saturated preform, and this showed that the temperature and degree of cure results as a function of time (that was validated against experimental DSC measurements). Deléglise et al. [25] implemented an algorithmic code using LIMS software to investigate the viscosity variations of a resin system during the mould filling process. In their study, the viscosity is only a function of time (isothermal—constant temperature) following an exponential form. This would allow the prediction of the viscosity–time relationship at any location of the fibre preforming throughout the filling stage. The flow patterns and simulations of a thin product part indicated a satisfactory match with experimental profiles. The study [25] placed emphasis on the use of a non-isothermal model rather than the developed isothermal solution, particularly in thick and complex shapes for the sake of a thorough resin characterisation. The commercial multiphysics tool, i.e., COMSOL, was employed by Sandberg et al. [43] to study the cure kinetics of polyurethane resin systems in a closed mould pultrusion process. The developed model included flow, thermal, and chemical equations, in which a contribution was made showing the possible initiation of curing from the pultrusion die centre because of the heated fibre preforms prior to the injection process. Despite the fact the coupled numerical scheme [43] with an ALE moving mesh approach successfully solved a convected flow problem, it was still bounded to regular geometries. Shi et al. [26] developed numerical formulations based on FE/CV method to calculate temperature variations and obtain cure kinetics (e.g., degree of cure) profiles during RTM process cycle. The study found the unstructured tetrahedron mesh more adaptable to complex shapes than the structured mesh, and also claimed that the 3D numerical simulation of such a complicated flow field gives more information than the 2D. Aktas et al. [44] attempted to simulate the cure behaviour and exothermic temperature during the vacuum infusion (VI) process cycle (fill and cure stages) using PAM-RTM software. The VI experiment was used to obtain filling and temperature profiles, while curing data were acquired by DSC test measurements. The numerical simulations showed good comparison, ∼3.92% error, for temperature results. However, the degree of cure predictions did not appear to be similar to the experimental ones (up to ∼80.4% error), as attributing the effects of fibre preform properties on cure simulation would yield different results from the DSC—involving only a resin sample. The flux-corrected transport was introduced by Tan and Pillai [4] to enhance the commercial PORE-FLOW FEM-based for modelling reactive flows in fibrous porous structures. This was to stabilise the FEM solution for the inclusion of energy and species (chemical) equations, and the work was validated against experimental data for temperature histories, albeit showing a time lag (4.8 s) between the compared results at the beginning of the mould filling—explained by the unevenness of the flow advancement. On that premise, resin kinetics and rheology simulations were performed on 3D porous architectures, highlighting that oscillations may occur in early stages.

Although some previous works have been reported on modelling convection dominated flows in LCM processes, further effort is required to address the cure kinetics and chemo-rheology effects on a resin flow during fill and cure stages as such studies are seldom. Most numerical studies found in the literature have focused on exothermic reaction (polymerisation) when the filling stage is completed. This means that the numerical algorithm would mainly solve energy-species equations to couple temperature and cure by the additional source terms.

**Figure 2:**
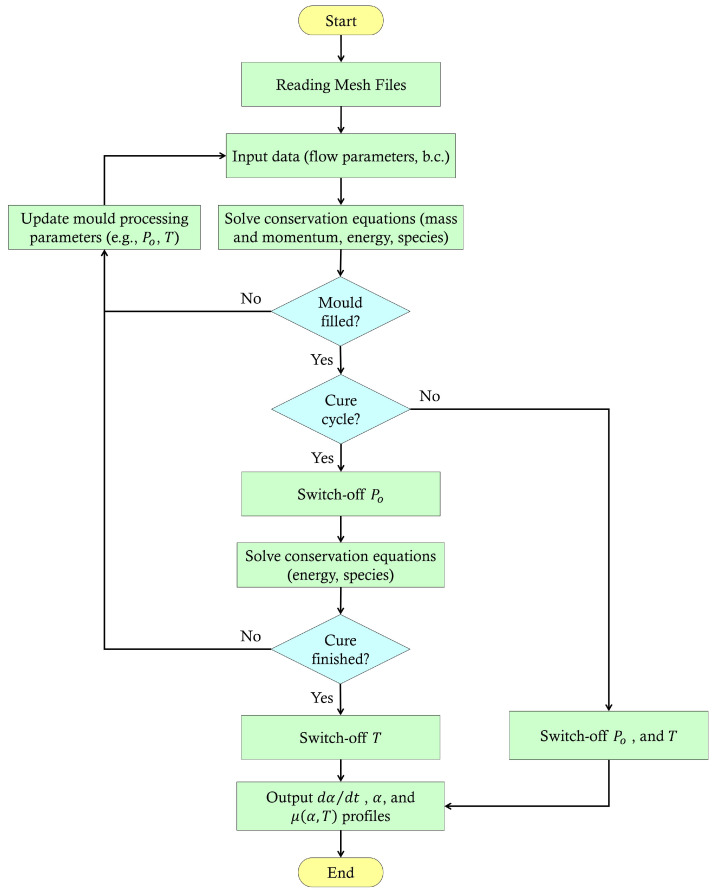
Flowchart that illustrates the proposed numerical approach for filling and curing the simulation of an LCM process cycle.

In the present research work, FVM/VOF is adopted by the present work to solve front-tracking, viscosity change, cure, and rate of cure—a thermo-chemo-flow model—of the liquid resin during mould-filling and post-mould-filling stage, unlike previous research that used FD/CV- or FE/CV-based schemes [4,20,21,22,23,24,25,26,41,42,43,44]. User-defined functions (UDFs) are created along with user-defined scalers (UDSs) and user-defined memory (UDM) to extend the ability of the standard ANSYS Fluent modules for modelling the cure, and chemo-rheology using “DEFINE” macros as illustrated in the Figure 2 flowchart. This further quantifies problem areas comprising irregular geometries and controls oscillations (error) for an accurate and more stabilised computational performance. The privileged use of a user-friendly numerical solution should be sufficient to optimise convection-dominated transport phenomena in LCM manufacturing processes as well as post-process and predict undesirable outcomes such as voids and degradation of the material.

## 2. Numerical Simulation

Flow simulation is solved by well-known Navier–Stokes (N–S) equations with a discretisation scheme based on the finite volume method (FVM). The volume-of-fluid (VOF) model is enabled to allow two-phase (e.g., resin, and air) flow modelling, and an implicit time-stepping scheme is adopted for greater stability in the computational process. In this numerical framework, a set of equations (thermo-chemo-flow) is computed to capture the cure kinetics, and chemo-rheological behaviour. Equations (4), (7), and (14) are written in C language (a source code) as UDFs (a custom function) and then loaded by a compiler together with the heat generation term in Equation (11). This is carried out using “DEFINE” macros to access data in a numerical solver. A flow chart explaining the solution scheme for a filling and curing simulation of an LCM process cycle is presented in Figure 2.

### 2.1. Flow Modelling

In the RTM process, Darcy’s law can be used to describe the resin impregnation through porous media. The filling process is simulated, assuming undeformed fabrics and a low Reynolds number $\left(R_{e}\ll 1\right)$, wherein inertia effects can be neglected. Since the viscous forces dominate the inertial forces (in the macroscopic level), the surface tension could also be ignored. Thus, the momentum flow equation can be represented by the three-dimensional Darcy model (see Equations (8) and (9)). This form follows an orthotropic fabric reinforcement, whereby the in-plane permeability (the ability of transporting fluids in porous materials) is denoted by $\left(K_{xx}=K_{yy}=K\right)$, while $K_{zz}$ stands for the out-plane (through thickness) flow.

(8) $$u=-\frac{1}{\mu }\left(K_{o}\cdot \nabla p\right)$$

(9) $$\left[\begin{array}{c} u_{x}\\ u_{y}\\ u_{z} \end{array}\right]=-\frac{1}{\mu }\left[\begin{array}{ccc} K_{xx} & 0 & 0\\ 0 & K_{yy} & 0\\ 0 & 0 & K_{zz} \end{array}\right]\cdot \left[\begin{array}{c} \partial p/\partial p\\ \partial p/\partial y\\ \partial p/\partial z \end{array}\right]$$

where $K_{o}$ is the global permeability tensor, μ is the viscosity of the fluid as a function of temperature and cure (T,α), and ∇p is the pressure gradient. It is worth mentioning that the global permeability $\left(K_{o}\right)$ is characterised by N–S/Brinkman equation that accounts for flows within open (inter-tow) and porous (intra-tow) regions, as elaborately discussed by Hwang and Advani [45] as well as in other related works by the latter [46,47,48,49]. This stems from the fact that the dual-scale nature (pores) of fabric structures impacts the resin-front motion during the mould-filling process. Such a difference in pore scales allows the swifter permeation of fluids in-between tows (meso-pore) than within tows (micro-pore), which can provoke voids or dry spots in the impregnated fibre preforms. The cure-temperature-dependent viscosity, as can be seen in Equation (7), is specified as a user-defined property function, during which the associated variables are passed by the numerical solver (i.e., ANSYS Fluent) to the user-defined-function (UDF)—a custom function to enhance the standard code—using “DEFINE_PROPERTY” macros for simulating a chemo-rheological behaviour. The flow-front advancement is considered in the continuity Equation (Equation (10)), in which the volume-averaged velocity u is defined as $u_{ij}/\phi =dL/dt$, ϕ is the medium porosity, *L* is the flow travel distance, and *t* is the time:

(10) $$\frac{\partial F_{i}}{\partial t}+\nabla \cdot \left(F_{i}u\right)=0$$

where $F_{i}$ represents the flow phase volume fraction defined by terms *a* for air and *r* for the liquid resin.

### 2.2. Heat Balance

The resin flow is affected by the mould temperature, whereby a convection-diffusion-type flow would be followed, leading to an exothermic reaction. This heat transfer phenomenon is quantified in the present work by the energy equation along with the heat generation rate term—a custom source term UDF defined by “DEFINE_SOURCE” macros that are called and passed into the function from the solver. The viscosity of a reactive resin system will initially decrease due to the increase in temperature; however, when the cross-linking/chemical reaction starts to take place, the viscosity would grow sharply attributed to the increasing extent of the cure. The commercial simulation software coupled with an implemented code taking the heat source term and viscosity evolution into account would be capable of modelling the set of thermo-chemo-flow equations. By solving this interaction (effect of temperature and curing on resin transfer), the important rheological behaviour of a complex flow could be attained during the mould filling process. A slow resin flow progression, which is usually the case for RTM and VARTM processes, provokes a small (approaching zero) Péclet number (e.g., 0.017)—∼0.54 Graetz number (Gz ≪ 1)—(owing to small velocity $∼9.52\times 10^{-5}\;\left[m/s\right]$), and hence that manifests the assumption of a local thermal equilibrium (i.e., fibre and resin share the same thermal properties), along with no contribution of dispersion into the heat flux term [21,41,50,51,52,53]. Thus, the energy equation can be expressed by Equations (11)–(13):

(11) $$\rho C_{p}\frac{\partial T}{\partial t}+\rho_{r}C_{p_{r}}\left(u\cdot \nabla T\right)=\nabla \left(K_{T}\cdot \nabla T\right)+\phi \rho_{r}\Delta H_{tot}\dot{G}\left(\alpha ,T\right)$$

(12) $$\left\{\begin{array}{c} \rho =\frac{\left(\rho_{f}\rho_{r}\right)}{\left(\rho_{f}w_{r}+\rho_{r}w_{f}\right)}\\ K_{T_{\|}}=w_{r}k_{r}+w_{f}k_{f},\;K_{T_{⊥}}=\frac{\left(k_{f}k_{r}\right)}{\left(k_{f}w_{r}+k_{r}w_{f}\right)}\\ C_{p}=w_{r}C_{p_{r}}+w_{f}C_{p_{f}} \end{array}\right.$$

(13) $$\left\{\begin{array}{c} w_{r}=\frac{\left(\phi /\rho_{f}\right)}{\left(\frac{\phi }{\rho_{f}}+\frac{1-\phi }{\rho_{r}}\right)}\\ w_{f}=1-w_{r} \end{array}\right.$$

where ρ, $C_{p}$, $K_{T}$, and *w*, are the density, heat capacity, thermal conductivity tensor, and weight fraction of the composite, respectively. Subscripts *f* and *r* stand for fibre and resin, respectively. $\Delta H_{tot}$ is the total reaction heat, and $\dot{G}\left(\alpha ,T\right)$) is the rate of reaction (cure). The thermal conductivity tensor shows diagonals $K_{T_{\|}}$ (in-plane) and $K_{T_{⊥}}$ (out-plane), and because of the symmetry in such an orthotropic material, the off-diagonal terms are set to zero [43,50]. The two terms on the left-hand side of Equation (11) show the transient and convection terms, while the two terms on the right-hand side represent the diffusion and heat generation terms, respectively.

### 2.3. Species Model

During the mould filling stage, the chemical equation can be employed using UDS (a defined scaler quantity—degree of cure—calculated by the solver using the transport equation and the supplied UDFs) to solve the curing kinetics as given in Equation (14). This expression ignores the molecular diffusion of macromolecule (diffusion) term, since the chemical reaction rate becomes much higher than the diffusion rate within the polymerisation process [20,26,41]. The formulation is also known as mass transfer or transport, and this defines the convection impact (u·∇α), and the change in the extent of the cure $\left(\frac{\partial \alpha }{\partial t}\right)$ at each point, in space and time, by virtue of “DEFINE_UDS_FLUX” and “DEFINE_UDS_UNSTEADY” macros, respectively. The rate of reaction is added as a source term to the species model, whereby the cure kinetics $\phi \dot{G}\left(\alpha ,T\right)$ can be coupled with the energy equation during the numerical simulation.

(14) $$\phi \frac{\partial \alpha }{\partial t}+\left(u\cdot \nabla \alpha \right)=\phi \dot{G}\left(\alpha ,T\right)$$

### 2.4. Boundary Conditions (b.c.)

Solving the conservation equations for mass and momentum, energy, and species requires a set of boundary conditions. At a resin inlet, a pore-averaged/volume-averaged resin pressure, temperature, and degree of cure are equivalent to the constant injection pressure, initial temperature of resin, and an assumed null initial degree of cure, respectively. Pressure vanishes at the resin-front, while this condition becomes complicated for temperature and cure effects as an exothermic heat generation occasion is brought-forth due to cure reaction rate and heat balance [4,26]. At a mould wall, the pore-averaged pressure is in a normal direction to an impermeable wall such that no resin progression emerges. A mould interface (resin/mould-wall-surface contact) yields roughly an equivalent volume-averaged temperature to the mould-wall temperature [4,26]. The employed b.c. while solving Equations (8), (10), and (14) are as follows:

(15) $$at\;inlet\;\to \left\{\begin{array}{c} p=p_{0}\\ T=T_{0}\\ \alpha =0 \end{array}\right.$$

(16) $$at\;flow\;front\;\to \left\{\begin{array}{c} p=0\\ \frac{d\left(F_{i}\rho C_{p}T\right)}{dt}=\frac{d}{dt}F_{i}\left(1-\phi \right)\rho_{f}C_{p_{f}}\left(T_{f}-T\right)+F_{i}\phi \rho_{r}\Delta H_{tot}\dot{G}\left(\alpha ,T\right)\\ \frac{d\left(F_{i}\alpha \right)}{dt}=F_{i}\dot{G}\left(\alpha ,T\right) \end{array}\right.$$

(17) $$at\;mould\;wall\;\to \left\{\begin{array}{c} \frac{\partial p}{\partial n}=0\\ T=T_{m} \end{array}\right.$$

## 3. Results

**Figure 3:**
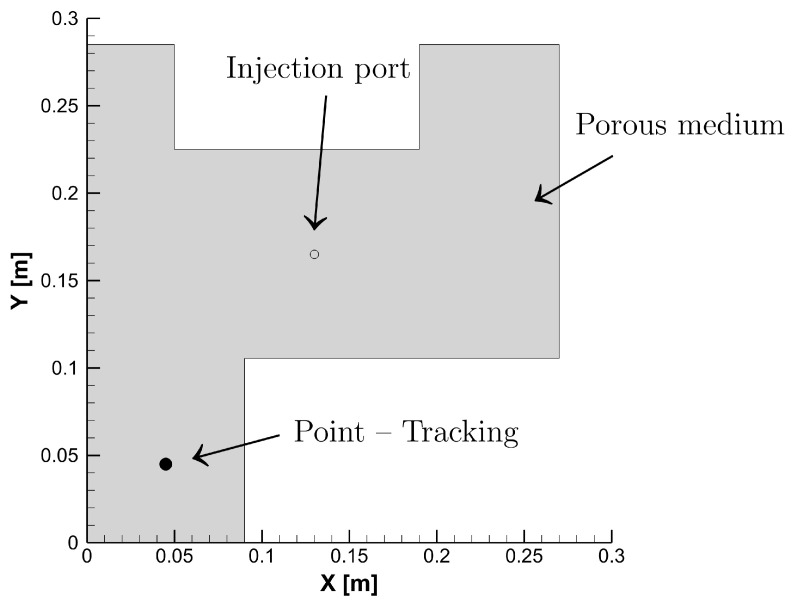
Part shape geometry and boundary conditions.

**Figure 4:**
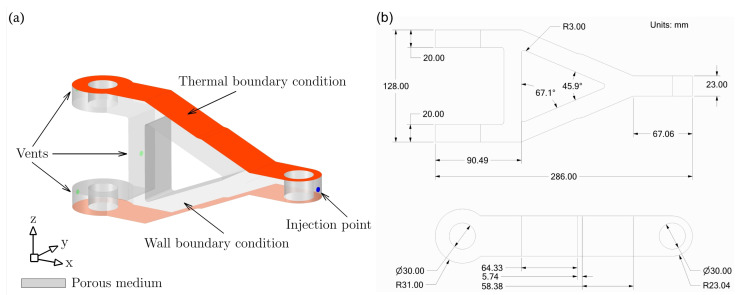
Composite torque link: (**a**) boundary conditions; and (**b**) geometric details.

**Figure 5:**
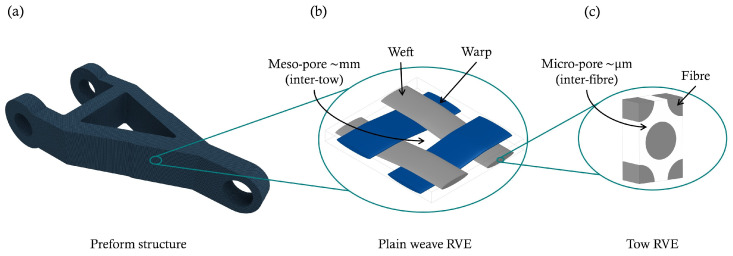
Multi-stage upscaling models and dual-scale pores in a preform structure: (**a**) global (macro) level; (**b**) tow level; and (**c**) fibre level.

Numerical simulations and customised UDF/UDS codes are performed using the commercial CFD software ANSYS-Fluent. This study applies the numerical approach on different three-dimensional geometric structures (c.f., Figure 3 and Figure 4), which should serve as a model (reflection/applicability) to regular and irregular composite shapes. The study also assumes undeformed (unsheared) fibre preforms during the filling process, and hence a homogeneous orthotropic porous medium is adopted. It is also important to note that dual-scale pores in fabric structures—see Figure 5—influence the advancing flow-front during the filling process, as the resin impregnation evolves intra- and inter-tow. This is therefore postulated in the given global permeability value, and such a phenomenon has been thoroughly discussed by the authors in previous related works [54,55]. The created mesh topology is, however, subject to the so-called mesh independence study (see previous numerical works [54,55] for further details), whereby controls for sizing (e.g., element size, growth rate, etc.) are required to refine the model until the independent results (e.g., velocity values) of the mesh resolution are reached—with no further effect of mesh on the simulation results. This is to avoid numerical diffusion (error) and to obtain a less computational expense and a valid solution, e.g., convergence with $10^{-5}$ or $10^{-6}$ residuals for energy and <1% net mass imbalance. The related fibre and resin properties, cure kinetics, and chemo-rheology parameters, are listed in Table 1 [24]. The results investigated the chemo-rheology and cure kinetics of the liquid resin. In the first part (Section 3.1) of the analysis, a comparative analysis was conducted to verify the present solution scheme with the work conducted by Shojaei et al. [24] for a simple composite shape. Then, in Section 3.2, a complex geometry is introduced to perform filling and curing simulations at various mould temperatures and study the cure kinetics and rheology of the resin flow in such designs.

### 3.1. Characterisation and Validation: A Simple Geometry

**Figure 6:**
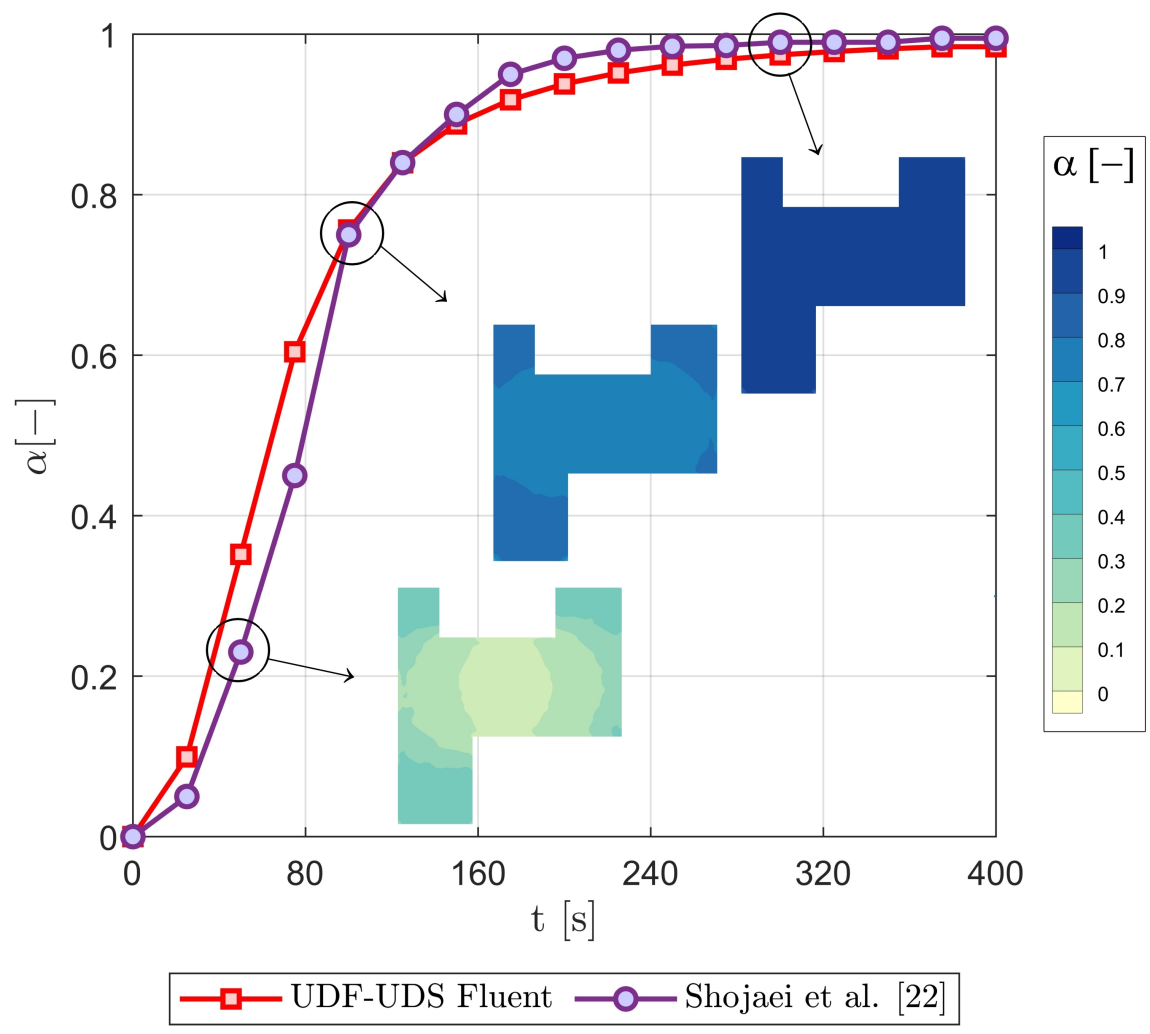
The degree of cure versus time at a mould temperature of 60 ${}^{\circ }C$.

In a chemo-rheological simulation, the viscosity behaviour is affected by the degree of cure and temperature; therefore, Equations (4) and (7) are used along with flow and thermo-chemical Equations (8), (10), (11) and (14). As mentioned earlier, the developed UDF and UDS are employed to incorporate the relevant equations and source terms. The finite volume method (FVM) is used by ANSYS-Fluent to convert the scaler transport equation to an algebraic form, wherein the scaler quantity “α” can be numerically solved. The degree of cure profiles can then be simultaneously reported during the numerical analysis, while the rate of the cure $\left(\frac{d\alpha }{dt}\right)$ history profile is initially stored and then retrieved using the user-defined memory (UDM). Simulations are performed on the same geometry of a fibrous porous medium with 8 mm thickness (c.f., Figure 3), at a constant injection pressure of 50 kPa, and with a bottom wall temperature of 60 ${}^{\circ }C$. These boundary conditions are taken from Shojaei et al. [24] together with the rheology and cure kinetics data. This is to compare and verify the current analysis with the FORTRAN CV/FEM-based model proposed by Shojaei et al. [24]. At the mould-wall (bottom-wall) temperature of 60 ${}^{\circ }C$ for any point that is not near the inlet, a verified analysis with Shojaei et al. [24] for the extent of cure vs. time history profile is presented in Figure 6.

**Figure 7:**
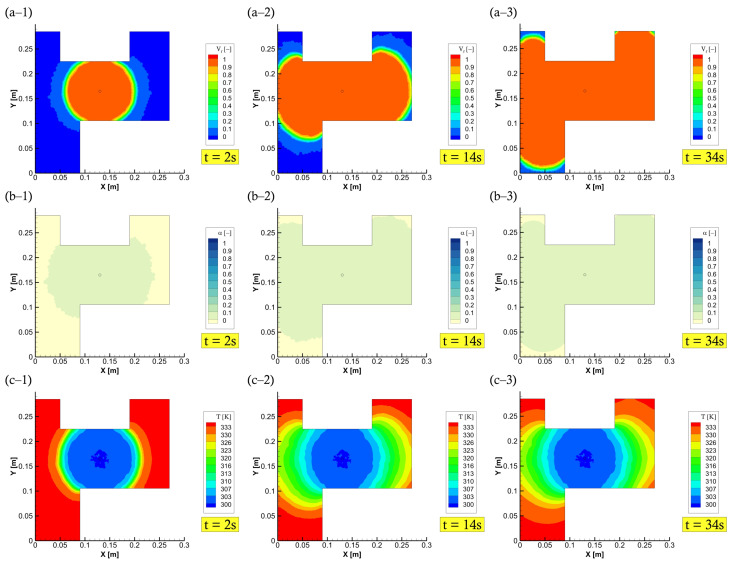
The simulation results related to the mould filling process: (**a**) resin impregnation and flow front; (**b**) degree of cure; and (**c**) temperature distribution.

This shows 0.026–3.4% difference between the two models for most of the reported values. The slight difference in results is attributed to the adopted numerical methodology. Other than the discretisation method, the set of governing equations is solved based on a single phase flow (a quasi-steady state formulation) by [24], while this is different for the current study that adopts a VOF-based two-phase flow solution. Thereby, the calculated and stored variables and properties in any computational cell would represent either fluid (resin) or air, or the mixture of the two phases (as can be seen in Figure 7a). Since such an interaction occurs in real composite manufacturing processes, the consideration of two-phase modelling is deemed to be more realistic. The profile plots (contours) of output data presented in Figure 7 (e.g., at 60 ${}^{\circ }C$ bottom-wall) explains that using such a coupling technique (linking flow/energy/species via UDF-UDS functions) on FVM/VOF-based solution, makes it possible to capture the curing temperature (includes bottom-wall and reaction heat sources) distribution during the resin impregnation (mould-filling) phase of textile preforms. In contrast to previous research [4,20,21,22,23,25,26,41,42,43,44], including [24], that did not place emphasis on such details.

### 3.2. Cure Kinetics and Chemo-Rheology Characterisation: A Complex Geometry

**Figure 8:**
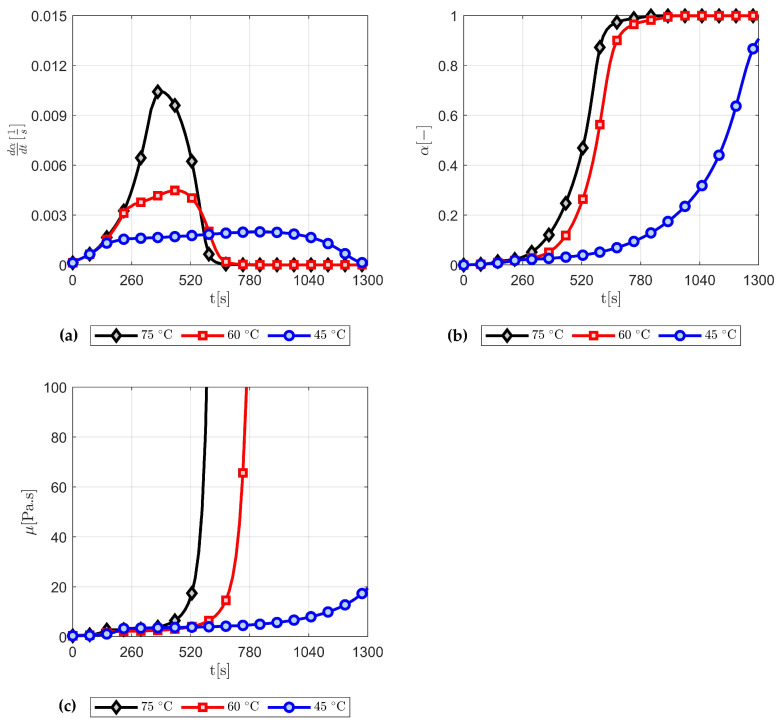
Numerical simulations of the reactive flows: (**a**) the rate of reaction; (**b**) degree of cure; and (**c**) viscosity evolution.

In this section, we present the numerical thermo-chemo-flow model for a complex structure, in particular, a real composite component product. An aerospace application, that is, a composite torque link is selected for an RTM numerical filling and curing simulation. A schematic of this torque link is presented in Figure 4 together with the related dimensions. In the aerospace industry, this torque link is used for helicopter landing gears, which has been a successful substitute for the traditional metal-based structure [56,57]. The processes, namely, RTM, VARTM, and autoclave, are utilisable and suitable means for manufacturing such a composite component [56,57]. The used resin and fibre preform properties in addition to the rheological and kinetic parameters of resin are the same as those presented in the previous sections—as can be seen in Table 1. The developed code coupled with the ANSYS-Fluent simulator via UDF, UDS, and UDM, shows its aptitude to localise the highly cured areas within the composite part during the filling stage, and this varies as per different mould temperatures. This also alludes to the fact that the evolution of the degree of cure (i.e., the progress of crosslinking reaction) affects the resin viscosity, and hence the gelling and curing of the liquid thermoset. The upper and lower mould plaques are heated with three various temperatures 45 ${}^{\circ }C$, 60 ${}^{\circ }C$, and 75 ${}^{\circ }C$. This is to investigate the impact of such a wall thermal boundary condition on the advancing resin flow in terms of viscosity, degree of cure, and rate of reaction. It is worth mentioning that this numerical simulation of the mould filling in the RTM process is non-isothermal, and a thermal equilibrium assumption for the solid and liquid phases is adopted—as can be seen in Equation (11). Since the geometry is a bit complex in shape, instead of default tetrahedral cells, the simulation domain is meshed using polyhedral cells. Polyhedral mesh is generally more suitable for complex geometries and convection-dominated flows with lowering computational expense and numerical diffusion, and allowing to use a different range of time step sizes [58]. This is in addition to the use of high-order upwinding interpolation schemes (e.g., second-order upwind scheme for the convection term or implicit modified high-resolution interface capturing (HRIC) scheme for VOF calculations) to attain better stability and convergence. Figure 8a shows the maximum rate of cure 0.0104 s${}^{-1}$, 0.0044 s${}^{-1}$, and 0.002 s${}^{-1}$ at 75 ${}^{\circ }C$, 60 ${}^{\circ }C$, and 45 ${}^{\circ }C$ mould temperatures, respectively. This occurs during the early stage of the mould filling and continues to apply over the post-filling stage (0–520 s), and that is due to the autocatalytic reaction mechanism being solved. Figure 8a also highlights the maximum curing rate that is swiftly achieved at higher temperatures, and this becomes slower and longer in time for lower curing temperatures. It is worth noting that such details (rate of cure) have not been numerically explored in the available literature, and hence, contributed by the present analysis—a developed UDM function to store and retrieve the rate of cure data over a computational runtime. In Figure 8b,c, the degree of cure and viscosity evolution profiles are presented. At the initial stage (0–520 s), and with 60 ${}^{\circ }C$ mould-wall temperature as an illustration, the curing reaction just begins due to the formation of the crosslink network that progresses. The crosslinking reaction (degree of cure) proceeds to transfer the resin system (phase change) from a viscous liquid to a rubbery state (520–910 s). As curing passes (>910 s) the gelation and the viscoelastic states, the thermoset solidifies and enters a glassy state to complete the cure process. As seen, a sharp viscosity increase occurred at a higher mould temperature, that is, at 75 ${}^{\circ }C$. This can be explained by the cross-linking reaction which is sensitive to extreme temperatures. Thereby, it causes fast curing at high temperatures, which solidifies the resin liquid faster.

**Figure 9:**
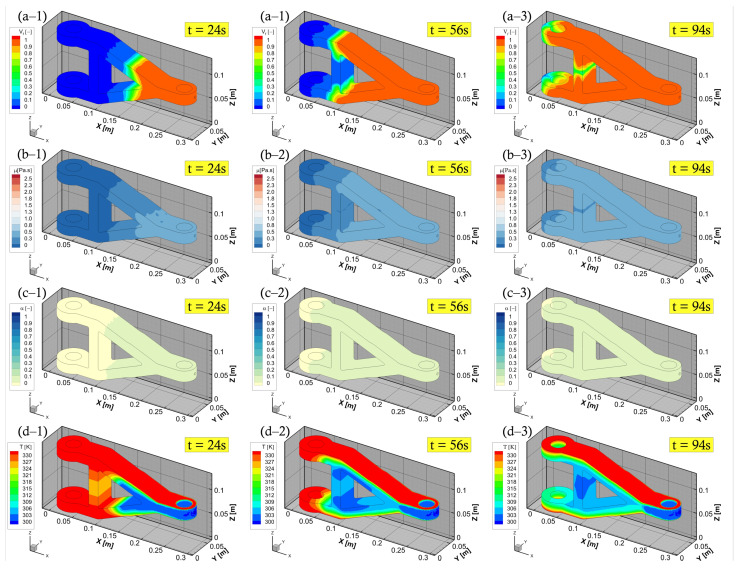
Filling stage of the torque link: (**a**) resin impregnation; (**b**) viscosity changes; (**c**) degree of cure; and (**d**) temperature distribution.

**Figure 10:**
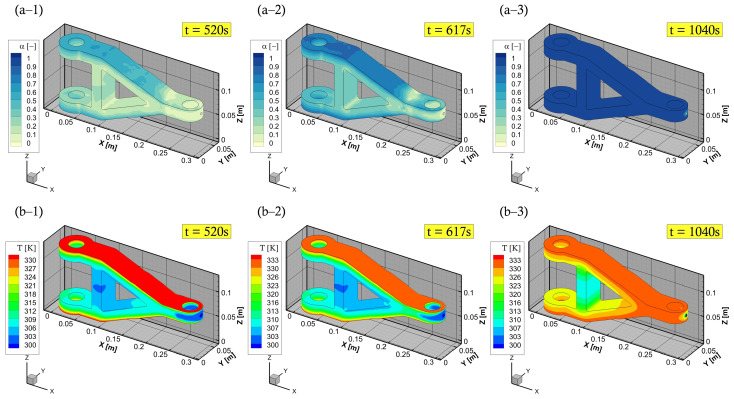
Cure stage of torque link: (**a**) degree of cure (cure finished); and (**b**) temperature distribution.

Herein, with a case at 60 ${}^{\circ }C$ mould temperature, the chemo-rheological observations are demonstrated in Figure 9, during the filling process stage. This shows a development overview of liquid resin advancement, degree of cure, temperature, and viscosity fields. In these plots, the degree of cure, during mould-filling, is observed to be low (less than the gel point—0.1) near the injection gate/port as well as through the fabric structure. It is stressed that a full saturation of the reinforced medium can be achieved at the aforementioned mould temperatures, and not reaching the gelation of such a thermoset. The developed numerical model is proficient to characterise these patterns, and allow varying process parameters (e.g., pressure, temperature, etc.) to control the viscous flow properties (below gel-point) and attain an optimal filling process. The numerical framework in this study also includes a command code for the inlet boundary condition, whereby the pressure injection is switched off whenever the mould is fully filled to only allow curing thereafter (c.f., Figure 10). This switching/shifting strategy can also be used for multiple gates’ injection to control (closed/open) resin transfer through inlet port/s, in addition to being applicable in the event of variable (dynamic) mould-wall temperatures. Consequently, providing a comprehensive competent tool that is diverse and attractive for LCM processes and the optimisation of highly convection-dominated flow problems.

## 4. Conclusions

Rapid cure can be undesirable, particularly during the resin impregnation process of fibre preforms. This is influenced by the temperature settings which accelerate the cross-linking reaction. To optimise convection–diffusion transport problems (reactive flows) in the LCM process, a set of thermo-chemo-flow models are developed and integrated into the standard solvers of the commercial CFD software ANSYS-Fluent using UDF, UDS, and UDM features. The numerical methodology approves its capability to simulate resin flows under variable process conditions. This allows the analysis of curing, the rate of cure, and viscosity during the filling process as well as when complete wet-out is obtained (post-filling). The model properly explains the developing chemistry as well as shows a good comparison with the corresponding results from the literature [24]. The multiphase flow method, i.e., VOF, is adopted by the current study in contrast to the CV/FEM-based quasi-steady state model in [24]. The flow and cure phenomena presented throughout the numerical simulations highlight the ongoing exothermic process (heat-generation)—e.g., reaching ∼4.97 $\times \;10^{7}\;J/m^{3}$ at 75 ${}^{\circ }C$ at both the resin impregnation and post-impregnation times of the fibrous porous media, as well as identify the time, temperature, and degree of cure relationship. This pre-processing or numerical prediction would be a worthy option to control, monitor online, and avoid the potential issues that may arise during the production of composite materials. With the broad applicability to regular and irregular geometries, and a variety of process technologies (including autoclave, pultrusion). Coupling of resin infusion and fibre deformation, in particular, chemical shrinkage and residual stress/strain would be next step to investigate. This will optimise impregnation, cure, and the subsequent structural integrity of the parts.

## Figures and Tables

**Table 1:** Material and process parameters [24].

Description	Parameter	Units
Resin and porous medium properties	$\rho_{r},\rho_{f}=1100,2560$	$\mathrm{kg}/m^{3}$
	$k_{r},k_{f}=0.168,0.0335$	W/(m·K)
	$C_{r},C_{f}=1680,670$	J/(kg·K)
Mould injection and flow process parameters	$p_{0}=50$	kPa
	$K_{xx}=K_{yy}=K_{zz}=2\times 10^{-9}$	$\;m^{2}$
	$\phi_{o}=60$%	—
	$V_{f}=40$%	—
Rheology and chemo-rheology parameters	$\mu_{0}=2.78\times 10^{-4}$	Pa·s
	$E_{\mu }=18,000$	J/mol
	R=8.3144	J/(mol·K)
	$\alpha_{gel}=0.1$	—
	a=1.5	—
	b=1	—
Cure kinetics	$A_{1}=3.7833\times 10^{5}$	$\;s^{-1}$
	$A_{2}=6.7833\times 10^{5}$	$\;s^{-1}$
	$E_{a_{1}}=54,418$	J/mol
	$E_{a_{2}}=50,232$	J/mol
	m=0.3	—
	n=1.7	—
	$\Delta H_{tot}=225\times 10^{3}$	J/kg

## Data Availability

Data are contained within the article.
